# Supplementary material for: Assessing corrosion resistance of two-dimensional nanomaterial-based coatings on stainless steel substrates
Source: R Soc Open Sci. 2020 Apr 29;7(4):200214. doi: 10.1098/rsos.200214 (PMC7211889; doi:10.1098/rsos.200214)
Supplement: Supplementary material from "Assessing Corrosion Resistance of 2D Nanomaterial-based Coatings on Stainless Steel Substrates" [file rsos200214supp1.docx]

**Assessing Corrosion Resistance of 2D Nanomaterial-based Coatings on Stainless Steel Substrates (RSOS-200214)**

**Shakir Bin Mujib, Santanu Mukherjee, Zhongkan Ren, and Gurpreet Singh**


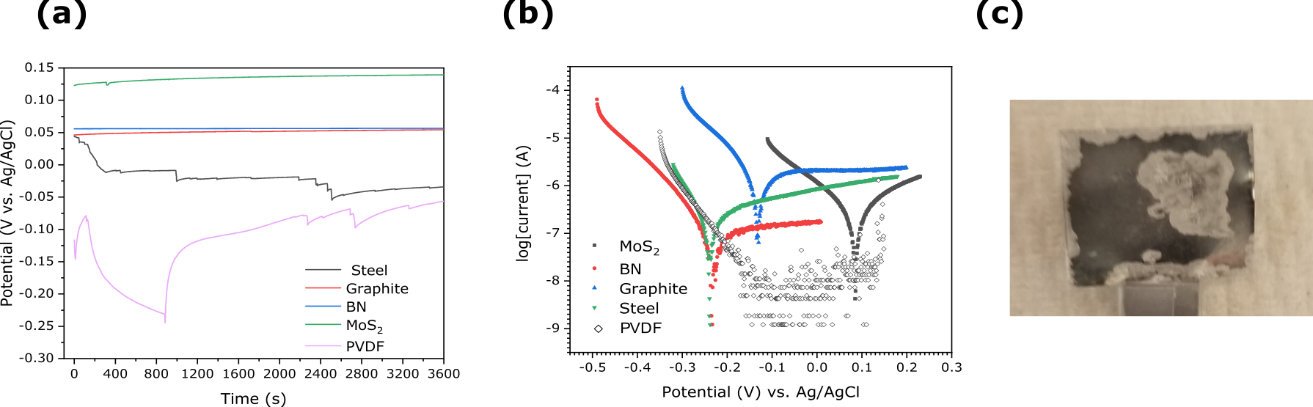


**Figure S1:** Performance of PVDF. (a) OCP, (b) Potentiodynamic polarization plot, and (c) digital image show that PVDF has poor resistance to corrosive environment in comparison with the coating materials.


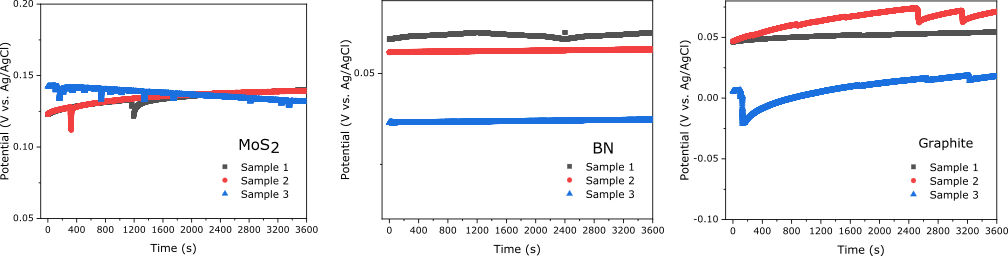


**Figure S2:** Comparison of OCP results of different samples in each coating.


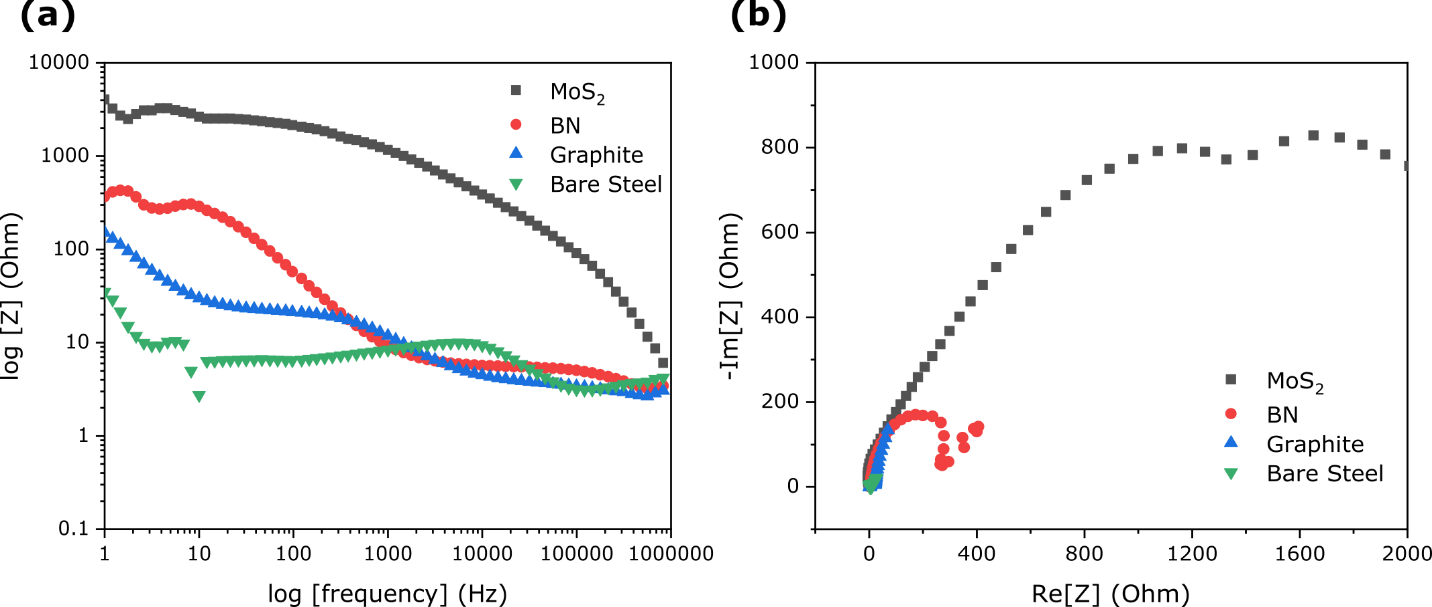


**Figure S3:** EIS test results of the pre-treated bare steel, exfoliated MoS_2_, BN and bulk graphite coated steel in 3.5 wt.% NaCl Solution. (a-b) A comparison between pre-treated bare steel, exfoliated MoS2, BN and graphite at the beginning of exposure (t = 0h). These plots clearly depict an order of magnitude difference in impedance values at the onset of exposure. Bare steel shows the lowest capacitive impedance indicating its poor resistance to corrosive environment.


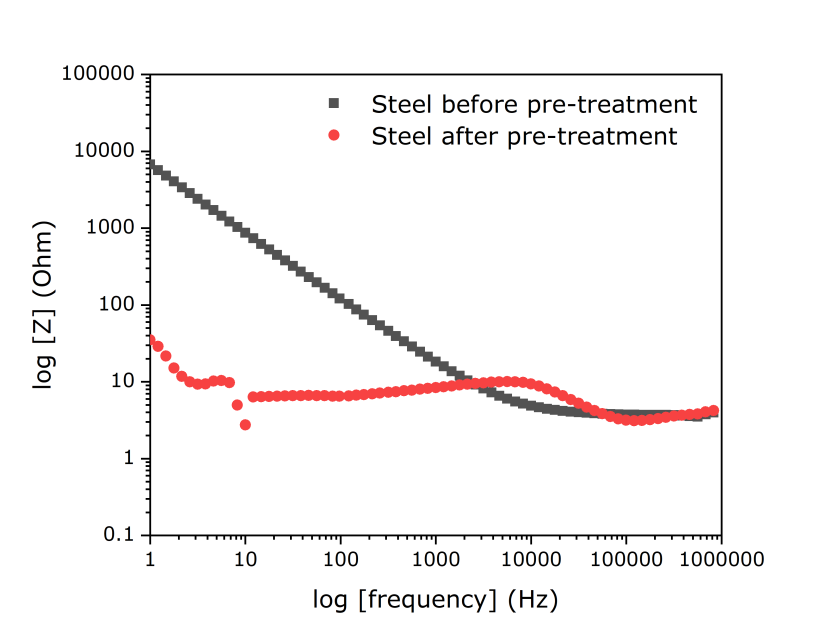


**Figure S4:** EIS test results of the bare steel, before and after the pre-treatment. The impedance value of the pre-treated sample went down suggesting the onset of corrosion on the steel.


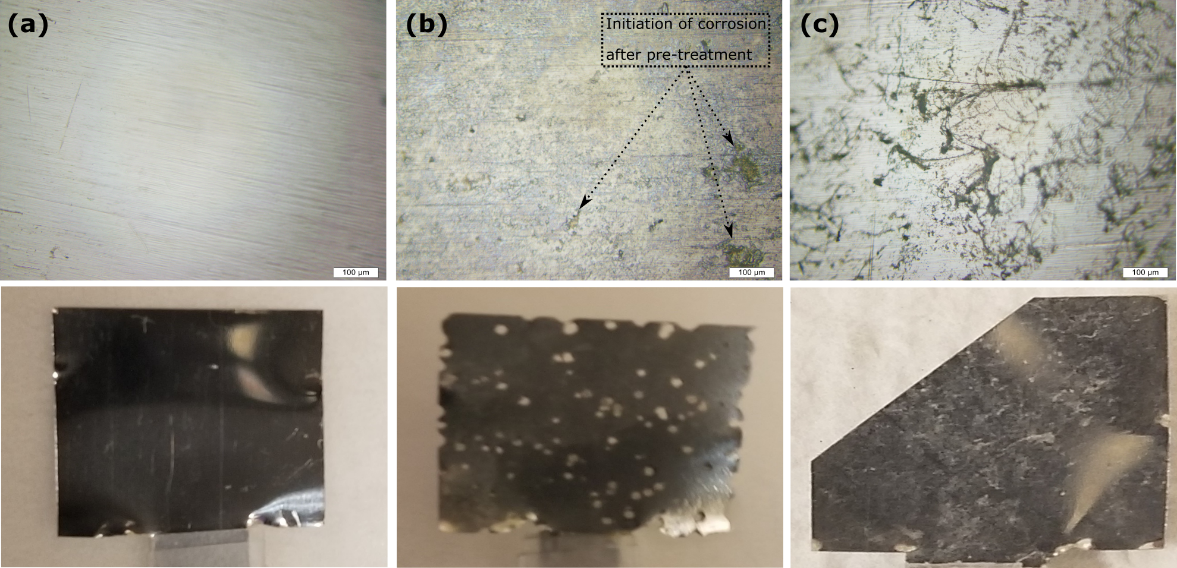


**Figure S5:** Optical and digital images of the (a) bare steel (before pre-treatment), (b) bare steel (after pre-treatment), and (c) bare steel (after long time exposure to NaCl) surfaces. The pre-treated sample shows the initiation of corrosion process on the steel after 5 days in 4M NaCl.
